# Dietary supplementation of silver-silica nanoparticles promotes histological, immunological, ultrastructural, and performance parameters of broiler chickens

**DOI:** 10.1038/s41598-021-83753-5

**Published:** 2021-02-18

**Authors:** Waleed M. Dosoky, Moustafa M. G. Fouda, Ali B. Alwan, Nader R. Abdelsalam, Ayman E. Taha, Rehab Y. Ghareeb, M. R. El-Aassar, Asmaa F. Khafaga

**Affiliations:** 1 Department of Animal and Fish Production, Faculty of Agriculture Saba Basha, University of Alexandria, Alexandria, 21531 Egypt; 2 Pre-Treatment and Finishing of Cellulosic Fabric Department, Textile Industries Research Division, National Research Center, 33 El-Behooth St., Dokki, Giza, 12311 Egypt; 3 Agricultural Botany Department, Faculty of Agriculture, Alexandria University, Saba Basha, 21531 Egypt; 4 Department of Animal Husbandry and Animal Wealth Development, Faculty of Veterinary Medicine, Alexandria University, Edfina, 22758 Egypt; 5 Plant Protection and Biomolecular Diagnosis Department, Arid Lands Cultivation Research Institute, The City of Scientific Research and Technological Applications, New Borg El Arab, 21934 Alexandria Egypt; 6 Polymer Materials Research Department, Advanced Technology and New Material Institute, City of Scientific Research and Technological Applications (SRTA-City), New Borg El-Arab City, 21934 Alexandria Egypt; 7 Department of Pathology, Faculty of Veterinary Medicine, Alexandria University, Edfina, 22758 Egypt

**Keywords:** Nanoscale materials, Soft materials, Cell biology, Genetics, Immunology, Zoology

## Abstract

Silver nanoparticles (AgNPs) have been used as a promising alternative to antibiotics in poultry feed. In this study, silver-doped silica nanoparticles (SiO_2_@AgNPs) were prepared in powder form, using starch, via the chemical reduction method and sol–gel technique followed by full characterization. SiO_2_@AgNPs were added to the poultry diet at three doses (2, 4, and 8 mg/kg diet). The safety of the oral dietary supplementation was estimated through the evaluation of the growth performance and hematological, biochemical, and oxidative parameters of birds. Moreover, the immunohistochemical examination of all body organs was also performed. Results of this study showed that SiO_2_@AgNPs have no negative effects on the growth performance and hematological, biochemical, and oxidative parameters of birds. Moreover, the immunohistochemical examination revealed the minimum inflammatory reactions and lymphoid depletion under a dose level of 8 mg/kg. In conclusion, SiO_2_@AgNPs could be considered as a promising and safe nano-growth promoter in broilers when added to poultry diet under a dose level of 4 mg/kg diet.

## Introduction

Since 2006, the European Union has prohibited the use of antibiotic growth promoters^1,2^. Thus, suitable alternatives to antibiotics are needed in poultry production. Recently, nanoparticles have been found to be novel alternative compounds due to their abilities to penetrate the intact physiologic barriers and reach several molecular targets^3,4^. Moreover, silver nanoparticles (AgNPs) have been also considered as one of the most promising components in several nanotechnology products^5–7^. The characteristic construction of the nanoscaled particles (1–100 nm), including silver nanoparticles, formed the main cause in their superiority over the macrostructures of the same chemical compound^8^, wherein the particle surface has a large number of atoms that are in direct contact with the external environment and has a direct effect on absorption properties. Conventionally, aqueous solutions of silver nanoparticles (AgNPs) deposited in various media, such as silica or polymers, have been used in agriculture sciences and animal production as a powerful sterilizing tool for equipment, storage places, and animal waste^9–11^. Currently, they are used as a novel feed additive in poultry farms, wherein they are expected to exert desirable effects on the immune status and growth performance of poultry due to their anti-inflammatory, antimicrobial, and immune-stimulatory activities. Meanwhile, some adverse impacts could be expected due to the induction of oxidative stress as an inherent reaction against the highly reactive nanosilver. Recently, few studies have investigated the safety aspect of the prophylactic use of nanosilver in poultry^9,12–14^. However, the results of these studies are still not sufficient to confirm the safety of the application of nanosilver in poultry.

Recent in vivo studies in quails and chicken embryos concluded that AgNPs have no adverse impact on growth^9^ and DNA oxidative damage in chicken embryos^9^. Moreover, they found that AgNPs exhibit a non-cytotoxic effect at a concentration of 0.1, 0.5, and 1.0%^15^ but exhibit a cytotoxic effect on human mesenchymal stem cells at a concentration of 2.5–50 μg/ml^16^. Some studies also came up with different conclusions regarding the ability of nanosilver to induce inflammation through the induction of reactive oxygen species (ROS) generation with the subsequent suppression of immune functions^17,18^, wherein other studies considered AgNPs as potent anti-inflammatory components of anti-inflammatory molecules^19,20^. It was well documented that colloidal silver nanoparticles are small enough to penetrate into the cell and subsequently into the nucleus and to directly interact with the nuclear DNA resulting in the modulation of gene expression profiles^21–24^, particularly genes related to inflammatory response (e.g., IL1β and TNF-α)^25^. Moreover, several studies stated that the concentration of Ag in broiler muscles could increase when the dose level of AgNPs is increased^26–28^; these nanoparticles may interact with proteins, resulting in biological and physiological changes in the cells^29^. Nabinejad et al.^30^ stated that the muscles and organs of the poultry may transfer the AgNPs to the consumers, leading to adverse effects. However, data regarding the safe application of orally administered AgNPs in poultry production is limited.

In this study, we hypothesized that the application of silver-doped silica nanoparticles (powder form) (SiO_2_@AgNPs) prepared using starch as a biopolymer and a natural polysaccharide in poultry production may potentially act as promising alternative to the use of antibiotic growth promoters. This study aims to investigate the safety margin of the oral application of SiO_2_@AgNPs in poultry production through the evaluation of growth performance, hematological and biochemical parameters, and immune and oxidative status of birds. Moreover, the histopathologic and immunohistochemical pictures of the liver, kidneys, spleen, thymus, and bursa of Fabricius were evaluated. The ultrastructure morphology and gene expression of IL1β and TNF-α were also determined in the muscle tissues of chickens.

## Materials and methods

The experimental procedures and methods were carried out following relevant guidelines and regulations. The Faculty of Agriculture Saba Basha, Alexandria University, approved all experimental protocols.

### Materials

Rice starch was purchased from yeast and starch Co. Egypt. Tetraethyl orthosilicate (TEOS) was purchased from Aladdin Reagent (Shanghai, China). Silver nitrate (AgNO_3_) was purchased from Merck Millipore, USA. Sodium hydroxide (NaOH) was purchased from AAT Bioquest Inc. (CA, USA). Other solvents, such as ethyl alcohol, were also used. The Milli-Q Type 1 Ultrapure Water System was used for the preparation and characterization of the nanoparticles.

#### Preparation of silver-doped silica nanoparticles (SiO_2_@AgNPs) in powder form

Firstly, 0.5 g of starch was dissolved in 100 ml of water and then 0.25 g of sodium hydroxide was added to the starch solution to reach a pH of 11. Moreover, a solution of silver nitrate (2%w/v) was added dropwise to the previous starch solution under continuous stirring. The temperature was raised to 70 °C. The obtained product was silver nanoparticles (AgNPs) and coded as solution A.

The sol–gel synthesis was outlined as follows: the sol was prepared by mixing the precursor tetraethyl orthosilicate (TEOS, 200 ml) with alcohol (400 ml) and water (85 ml). The mixture of ethanol–water was added dropwise to avoid the rapid hydrolysis of the precursor. After the complete addition of the co-solvent, the ready sol-solution was coded as solution B. Moreover, solution B was added dropwise to solution A under continuous stirring. Ammonia was then added to precipitate the formed SiO_2_ and AgNPs together. The solution was subjected to filtration and the precipitate (PPT) was washed several times with an excess amount of water to remove residual NH_4_OH and unreacted TEOS. The end product was silver-doped silica nanoparticles or silver-silica nanoparticles in powder form that were coded as SiO_2_@AgNPs.

#### Characterization of the formed silver-doped silica nanoparticles (SiO_2_@AgNPs)

To examine the prepared sample with the Transmission electron microscope (TEM), the sample of SiO_2_@AgNPs was deposited in on a “carbon-coated copper grid” and was left for drying at room temperature followed by the characterization through the *TEM instrument* (JEOL 200 kV, Japan). The *Nano-Sizer SZ90* (Malvern instruments Ltd., UK) was used to evaluate the “particle size” and the “zeta potential” of the formed SiO_2_@AgNPs in powder form. The size distribution and zeta potential of the as prepared SiO_2_@AgNPs were calculated at a pH of 7 and a temperature of 25 °C. Scanning electron microscopy (SEM) (JEOL, JSM-6360LA, Japan) was used to illustrate the internal structure and surface morphology of the SiO_2_@AgNPs. X-ray diffraction (XRD) analysis was performed to check the crystallinity and the specific peaks of the formed SiO_2_@AgNPs via the “XRD sate of art Panalytical Emperian, Turkey” pertaining to the CuKa radiation and was operated with a power of 40 kV and a 2-theta range of 10–80.

### Experimental design

The present experimental work was performed at the Poultry Research Laboratory, Animal and Fish Production Department, Agricultural Botany Department, Faculty of Agriculture (Saba Basha), Department of Pathology, Faculty of Veterinary Medicine, Alexandria University, in cooperation with the National Research Centre, Dokki, Cairo, Egypt. This study was conducted from July to August 2019. A total of 288 1-day-old male Cobb chicks were purchased from a local commercial hatchery with an average weight of 42.6 g. The chicks were randomly assigned into 12 pens (1.35 m × 1.45 m) in an open-sided house (4 treatments × 3 replicate × 24 chicks)^31^. Natural ventilation through the windows created inside the house was used. The temperature inside the house started at 33 °C at Day 1 and gradually reduced until it reached 31 °C after 2 weeks of age. Humidity ranged from 57 to 77% RH inside the house. Feed and water were offered ad libitum. The chicks were vaccinated against the Newcastle disease virus (NDV) using the HB1 strain combined with infectious bronchitis (IB) using the IB 120 strain at Day 7 and the LaSota strain only at Day 21. They were also vaccinated against infectious bursal disease using the D78 strain at Day 13.

The chicks were fed with a starter diet from Day 1 to Day 28 and a growing diet from Day 29 to Day 35^32^ (Table 1). The experimental diets were formulated according to the feeding stage: 23% of corn/soybeans in the starter diet (and standard protein requirement according to Nation Research Council (NRC)^33^ and 21% of crude protein in the growing diet. The samples from each starter and growing diets were randomly collected for proximate analysis in accordance with the procedure described by the Association of Official Analytical Chemists^34^.

**Table 1:** Composition and calculated analysis of the basal experimental diets.

Ingredients (%)	Starter	Grower
Yellow corn	55.750	59.590
Soybean meal (48%cp)	38.000	33.150
Sun flower oil	2.000	3.000
Mono calcium phosphate	1.600	1.600
Limestone	1.600	1.650
Sodium chloride	0.300	0.300
Vit. and mineral mix^a^	0.300	0.300
dl-Methionine	0.210	0.210
Lysine	0.200	0.200
Total	100.00	100.00
**Calculated analyses**^**b**^		
Crude protein (%)	22.98	20.98
ME (kcal/kg)	3004	3104
Crude fat (%)	2.50	2.60
Crude fiber (%)	2.71	2.60
Calcium (%)	0.99	1.00
Phosphorus available (%)	0.49	0.48
Mathionine (%)	0.57	0.48
Mathionine + cysteine (%)	0.84	0.83
Lyine (%)	1.37	1.25

^a^Each kg of vitamin and minerals mixture contained: Vit. A, 4,000,000 IU; Vit. D3, 500,000 IU; Vit. E, 16.7 g.; Vit. K, 0.67 g.; Vit. B1, 0.67 g.; Vit. B2, 2 g.; Vit. B6, 0.67 g.; Vit. B12, 0.004 g.; Nicotinic acid, 16.7 g.; Pantothenic acid, 6.67 g.; Biotin, 0.07 g.; Folic acid, 1.67 g.; Choline chloride, 400 g.; Zn, 23.3 g.; Mn, 10 g.; Fe, 25 g.; Cu, 1.67 g.; I, 0.25 g.; Se, 0.033 g.; Mg, 133.4 g.

^b^Calculated values were according to NCR^33^ textbook values for feedstuffs all the experimental diets were formulated to meet or exceed the National Research council recommendation (NRC)^33^.

The dietary treatments were as follows: chicks in the control group were fed with basal diets without any addition and those in the other groups were fed with basal diets supplemented with 2, 4, and 8 of nano-silica-silver mg/kg, respectively.

All of the chicks were reared in wire batteries under the same managerial, hygienic, and environmental conditions^35^. They were exposed to 23 h of continuous light per day during the experimental period. Feed and water were available ad libitum throughout the 6-week experimental period. Bodyweight and feed consumption were recorded. The feed conversion ratio was calculated (g feed/g gain). At the end of the experiment, blood samples were collected from the brachial veins of four chicks randomly chosen from each group. Serum was immediately centrifuged at 3500 rpm for 15 min and stored at − 18 °C until use.

### Evaluation of growth performance parameters

Live body weight, body weight gain, feed consumed, and feed conversion ratio (g feed:g gain) were recorded at 35 days of age for each replicate. No mortality was recorded during the whole experimental period.

#### Physiological measurements

At 5 weeks of age, six chickens from each treatment were randomly taken and slaughtered, and blood samples were collected and divided into two equal parts: the first part was collected on heparin as anticoagulant (0.1 ml of heparin to 1 ml of blood) according to Hawk et al.^36^ to determine the blood hematology (white blood cell counts (WBCs) and the differential of white blood cells, red blood cells (RBCs), hemoglobin concentration (Hb), and packed cell volume (PCV)), and the second part was immediately centrifuged at 3500 rpm for 15 min and stored at − 18 °C until use.

All biochemical analyses (total protein, albumin, alanine aminotransferase (ALT), aspartate aminotransferase (AST), alkaline phosphatase, uric acid, creatinine, calcium, phosphorus, silica-silver, total lipids, cholesterol, low-density lipoprotein, high-density lipoprotein, triglycerides) were performed using the commercial kits produced by Biodiagnostic, Egypt (www.bio-diagnostic.com).

### Evaluation of immunoglobulin M and G

Serum immunoglobulin (Ig) fractions were determined, according to Mancini et al.^37^. Phagocytic activity was established, according to Kawahara et al.^38^. A 50 μg of *Candida albicans* culture (previously adjusted to 1 g *Candida albicans*/100 ml saline) was added to 1 ml of citrated blood collected from the infected and control groups and shaken in a water bath at 23–25 °C for 3–5 h. Smears of the blood were stained with Giemsa solution. Phagocytosis was estimated by determining the proportion of macrophages that contained intracellular yeast cells in a random count of 300 phagocytes and expressed as percentage of phagocytic activity (PA). The number of phagocytized organisms was counted in the phagocytic cells, and the phagocytic index (PI) was measured

$$\begin{aligned} & {\text{Phagocytic activity}}\left( {{\text{PA}}} \right) = {\text{Percentage of phagocytic cells containing yeast cells}}. \\ & {\text{Phagocytic}}\;{\text{index}}\left( {{\text{P}}.{\text{I}}.} \right) = \frac{{{\text{number}}\;{\text{of}}\;{\text{yeast}}\;{\text{cells}}\;{\text{phagocytized}}}}{{{\text{number}}\;{\text{of}}\;{\text{phagocytic}}\;{\text{cells}}}} \\ \end{aligned}$$

At 3 weeks of age, all birds were vaccinated against the NDV using the HB1 strain. Blood samples (four samples from each treatment) were collected at 14 days after vaccination and immediately centrifuged at 4000 rpm for 15 min to separate serum. The hemagglutination inhibition test was used to determine the humoral antibody titer against the NDV.

### Lymphoid organ weight and some carcass traits

At the end of the experimental period, six birds from each dietary treatment were randomly taken, had fasted for 6 h then weighed and slaughtered to complete bleeding, and weighed to determine the relative weight of the immune organs (spleen, bursa, and thymus gland)^39^.

### Evaluation of oxidant/antioxidant parameters

Serum total antioxidant capacity, catalase, and malondialdehyde were analyzed using the commercial kits produced by Biodiagnostic, Egypt (www.bio-diagnostic.com), in accordance with the method of Motor et al.^40^.

### Gene expression

The RT-PCR was used to determine the specific expression of the different genes. Overall, TRIzol reagent (Invitrogen, Life Technology, Carlsbad, CA, USA) and NanoDrop for quantification have been used to extract a total RNA of around 100 mg from the muscle tissues. For the synthesis of DNA using a cDNA synthesis kit (Fernmentas, Waltham, MA, United States), A260 or A260/A280 RNA samples were used.

### Histopathologic evaluation

Immediately after slaughtering, tissue specimens were collected from the liver, kidney, spleen, thymus, and bursa from control and SiO_2_@AgNPs—treated chickens. Collected specimens were fixed in neutral-buffered formalin solution (10%) for 48 h. Following, fixed specimens were routinely processed via the conventional paraffin-embedding technique as previously described^41^. Several 5-μm-thick sections were microtomed and stained with haematoxylin and eosin (H&E). Tissues were blindly examined, evaluated, and captured by experienced pathologist (AFK). Representative photomicrographs were obtained with a digital camera (Leica EC3, Leica, Germany) connected to a microscope (Leica DM500).

### Immunohistochemical evaluation

From each paraffin block, additional 4 μm-thick sections were obtained, deparaffinized in xylene, rehydrated in a descending grade of ethyl alcohol, and retrieved for antigens through citrate-buffered saline (0.01 mol/l; pH 6.0). Following, the endogenous peroxidase activity was depleted by H_2_O_2_ in phosphate-buffered saline [0.3% (v/v)]. Then, samples were incubated with 10% (v/v) normal goat serum for one hour to block the non-specific immunologic binding. After that, sections were incubated overnight at 4 °C with mouse anti chicken monoclonal CD45 (MCA2413GA; Bio-Rad laboratory, Athina, Greece). Following that, sections were washed with PBS, incubated with biotin-conjugated goat anti-mouse IgG antiserum (Histofine kit, Nichirei Corporation, Japan) for 60 min, washed again with PBS, and incubated for 30 min with streptavidin-peroxidase conjugate (Histofine kit, Nichirei Corporation, Japan). The visualization of streptavidin–biotin complex was performed with 3,3′-diaminobenzidine tetrahydrochloride (DAB)-H_2_O_2_ solution (pH 7.0, for 3 min). Finally, sections were counterstained with Mayer's haematoxylin solution and blindly examined and captured by an experienced pathologist (AFK) using digital camera (Leica EC3, Leica, Germany) connected to a microscope (Leica DM500).

### Ultrastructure evaluation of muscle tissues

For ultrastructure examination, small specimens were immediately collected from breast muscle after dissection, tissues were cut into small pieces (about 1 mm^3^) and fixed for 3 h in 3% glutaraldehyde solution (Merck, Darmstadt, Germany) in 0.1 M sodium phosphate buffer (pH 7). Following, specimens were washed in two consecutive changes in buffer and then moved to a 1% osmium tetroxide solution (Electron Microscope Science, Sigma-Aldrich) for 1 h in 0.1 M phosphate buffer (pH 6.9). After that, tissue samples were washed for 5 min in 0.1 M sodium phosphate buffer, dehydrated in ascending grade of ethyl alcohol, and impregnated with Epon embedding resin. Then, samples were embedded and blocked at 60 °C for 48 h. Semi-thin sections were cut from the prepared blocks and stained with 1% basic Toluidine blue for the light microscopy. Following, ultrathin sections (50–80 nm) were prepared from the selected regions and moved onto copper grids (200 meshes). Finally, uranyl acetate dihydrate (2%) and lead citrate were used for sections contrasts. Tissues were examined and captured blindly by experienced pathologist (AFK) using JEM-1220 TEM (JEOL, Tokyo, Japan), with a Morada 11 megapixel camera (Olympus Soft Imaging Solutions GmbH, Münster, Germany).

### Statistical analysis

The differences among groups were statistically analyzed by one-way ANOVA using SPSS statistical software package for windows version 11.0. The significant variation inter group means were detached by Duncan's Multiple Range-test^42^.

## Results

### Synthesis of silver-doped silica nanoparticles (SiO_2_@AgNPs)

This study was designed to prepare more metal nanoparticles in one pot synthesis. Firstly, silver nanoparticles (AgNPs) were environmentally prepared using starch, a huge molecular weight carbohydrate polymer. One of the advantages for using starch as a stabilizing agent, aside from its potential power to convert the ions of silver to zero-valent silver, is the presence of huge hydroxy groups that stabilize the formed nanoparticles in nanoform. In our published work^7,28,43–47^, the prepared nanoparticles were extensively characterized using advanced tools. Secondly, as mentioned in the experiment part for the preparation of SiO_2_NPs, condensation reaction was performed for this synthesis. Condensation is one of the common ways for the modification purpose, owing to homogeneous incorporation of AgNPs in the interior of the bulk of silica particles. Thus, we aimed to exploit these nanoparticles to act as an embryo and shell for AgNPs (SiO_2_@AgNPs). Before the application of the fabricated SiO_2_@AgNPs, it is necessary to characterize it in detail using the TEM, dynamic light scattering (DLS), zeta potential, and SEM–EDX tools.

**Figure 1:**
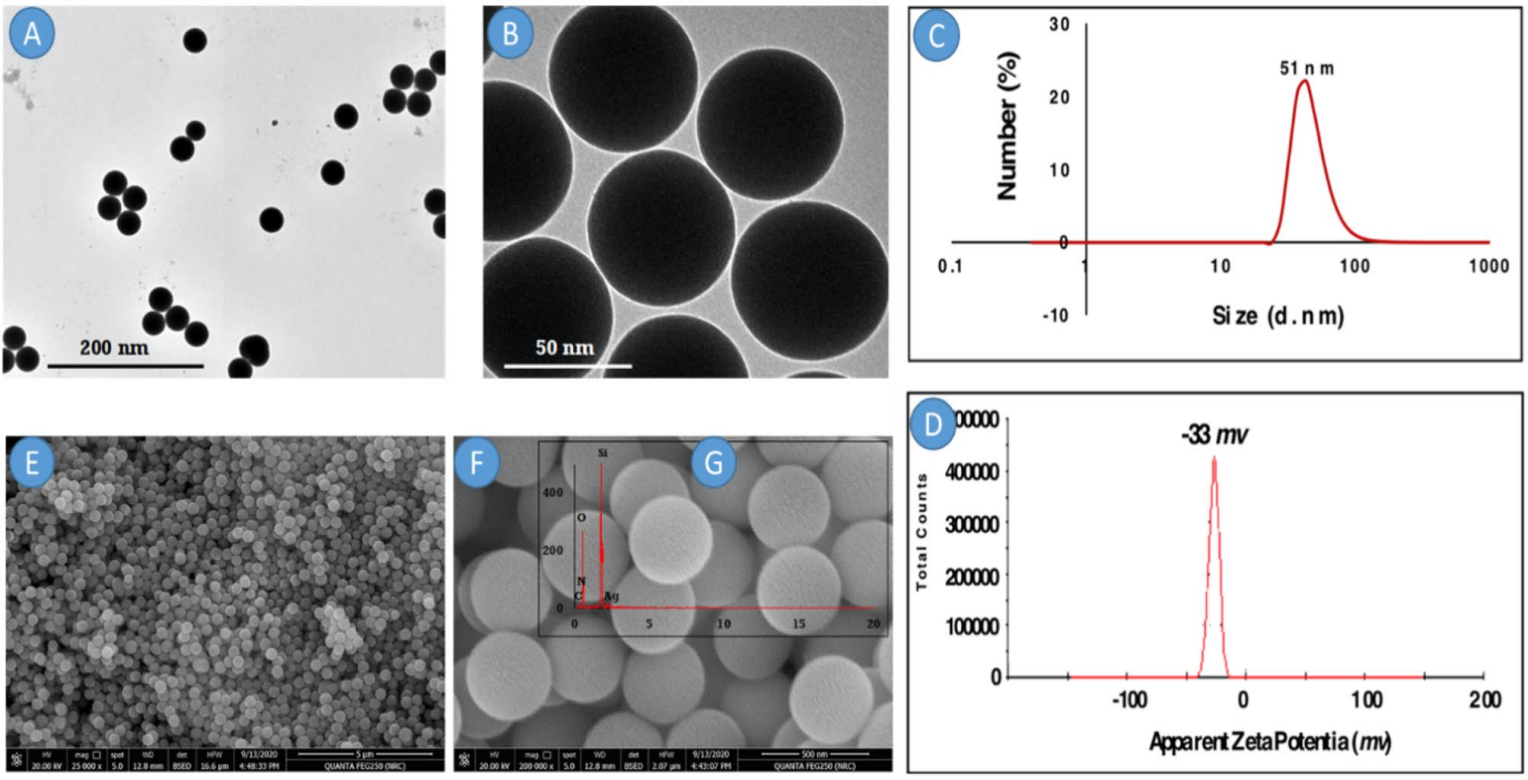
TEM of SiO_2_@AgNPs at (**A**) low magnification and (**B**) high magnification and (**C**) hydrodynamic size and (**D**) apparent zeta potential of SiO_2_@AgNPs and (**E**) SEM of SiO_2_@AgNPs at low magnification and (**F**) high magnification and (**G**) EDX of SiO_2_@AgNPs.

Firstly, the TEM instrument was used to characterize the shape of the resultant SiO_2_@AgNPs. It was clearly observed that SiO_2_@AgNPs (Fig. 1A,B) had a spherical shape and uniform particle size. Moreover, the average diameter of the spherical particle was around 20 nm. The onset image was taken at a high magnification power to clarify the particle shape of silver-doped silica nanoparticles. It can be concluded that SiO_2_NPs have a spherical shape with a porous structure. Due to the presence of AgNPs, these pores were filled with AgNPs. It was clearly observed that the prepared particles were well spherical with monodispersity. Moreover, it should be noted that the particles of included AgNPs were homo-distributes that appeared as a white light spot (Fig. 1B).

Figure 1C, represents the hydrodynamic size of SiO_2_@AgNPs, it is revealed that the average hydrodynamic diameter of SiO_2_@AgNPs is 51 nm with the polydispersity index (PDI) below 0.24 (below 0.5) affirming that, the nanoparticles possess relatively narrow size distribution and well dispersibility. In addition, SiO_2_@AgNPs has negative zeta potential (− 33 mv) due to the presence of stabilizing agent stabilized the resulted nanoparticles from agglomeration. As shown in Fig. 1D, the zeta potential of SiO_2_@AgNPs is less than 30 mV (− 33 mv) indicating the good stability of these nanoparticles in aqueous solution.

The surface structure and morphology of SiO_2_@AgNPs was investigated using SEM. Figure 1E,F displays the morphological structure of SiO_2_@AgNPs at two magnifications (25,000× and 200,000×) respectively. The SiO_2_@AgNPs is fairly uniform spherical particles with an average size of 150–250 nm. It is remarkable that the particles are formed with sufficient aggregation which could be attributed to by the polydispersity of the sample and indirectly confirms the presence of aggregate particles.

The examination of the particle shape of the resultant nanoparticles via the TEM instrument was performed for the sample after placing it onto the copper-coated grid. Thus, the particle does not have the tendency to agglomerate during the measurement. Hence, the particles were formed with a nearly small size when compared with the DLS data. The latter needs the evaluated sample to stand in the instrument for a long time in a solution. Therefore, the particles in the solution tend to aggregate into large particles.

Therefore, spherical SiO_2_@AgNPs are formed due to isotropic growth. Briefly speaking, the anisotropic structures of SiO_2_@AgNPs could be induced by growth rates on different directions at low temperature. On the contrary, the high temperatures resulted in the production of non-spherical particles and lead to severe anisotropic growth, resulting in the formation of rod-like particles with tetragonal cross section (data not shown). Moreover, the presence of AgNPs in the reactants has no considerable effect on the morphology of SiO_2_NPs. Thus, it can be confirmed that the morphology of SiO_2_@AgNPs mainly depends on the reaction temperature. Based on the onset image (Fig. 1G) (elemental analysis of the scanned sample via EDX), it should be noted that the sample contained four elements: carbon, oxygen, silicon, and silver. The presence of carbon and oxygen is attributed to the existence of natural polymers (starch) and oxygen connected to silica nanoparticles. On the other hand, the existence of Si and Ag affirmed the formation of SiO_2_@AgNPs.

### Effect of SiO_2_@AgNPs on the growth performance parameters

Table 2 shows the effect of the dose levels of SiO_2_@AgNPs on the growth performance of boiler chickens. No significant difference in the live body weight (g) and body weight gain (g) of the boiler chickens was observed in relation to the increasing dose levels of SiO_2_@AgNPs at all ages. While there are different between the SiO_2_@AgNPs as numbers between values, for instance after 21 days 2 mg/kg diet SiO_2_@AgNPs level recorded the highest body weight of boiler chickens (911.99 g) comparing with other two levels and control which. At 35 days, the body weight was 1952.50 g under a dose level of 8 mg/kg and increased by 33.20 g when compared with the control group (Table 2). The body weight gain increased by 869.32 g for the period of 1–21 days under a dose level of 2 mg/kg and increased by 1059.60 and 1919 g under a dose level of 8 mg/kg for the period of 21 to 35 days. The body weight gain increased by 33.40 g when compared with the control group (Table 2). The results showed no significant effect for the feed consumption (g/bird) and feed conversion ratio (g feed/g weight gain) between treated and control birds. Moreover, the FCR recorded the lowest non-significant values (P > 0.05) when compared with the control group, for instance, 1.410 g (g feed/g weight gain) in the control group and 1.383 g (g feed/g weight gain) under a dose level of 8 mg/kg for 1–35 days (Table 2).

**Table 2:** Effect of different levels of (SiO_2_@AgNPs) on productive performance, hematological and immunological parameters of boiler chickens from 1 to 5 weeks of age (n/group = 24).

Traits	(SiO_2_@AgNPs) levels (mg/kg diet) (means ± SD)				
	0	2	4	8	
**Body weight (g)**					
1 day	42.73 ± 3.39	42.67 ± 2.92	42.54 ± 2.81	42.50 ± 3.21	
21 days	881.66 ± 60.52	911.99 ± 61.18	891.26 ± 64.86	892.94 ± 104.46	
35 days	1919.30 ± 179.08	1894.60 ± 144.66	1931.10 ± 182.12	1952.50 ± 215.64	
**Body weight gain (g)**					
1–21 days	838.93 ± 61.22	869.32 ± 60.80	848.72 ± 66.37	850.43 ± 103.97	
21–35 days	1037.60 ± 143.56	982.60 ± 95.61	1039.90 ± 136.73	1059.60 ± 158.89	
1–35 days	1876.60 ± 180.13	1851.90 ± 144.12	1888.60 ± 183.31	1910.00 ± 216.22	
**Feed consumption (g/bird)**					
1–21 days	1107.10 ± 27.74	1125.00 ± 7.74	1125.00 ± 36.13	1118.90 ± 8.64	
21–35 days	1533.10 ± 21.39	1515.20 ± 1.40	1534.20 ± 26.81	1521.40 ± 8.64	
1–35 days	2640.20 ± 17.35	2640.20 ± 4.35	2659.20 ± 18.63	2640.20 ± 6.63	
**Feed conversion ratio (g feed/g weight gain)**					
1–21 days	1.320 ± 0.02	1.295 ± 0.02	1.328 ± 0.06	1.315 ± 0.01	
21–35 days	1.485 ± 0.13	1.545 ± 0.05	1.478 ± 0.03	1.443 ± 0.10	
1–35 days	1.410 ± 0.06	1.428 ± 0.03	1.413 ± 0.04	1.383 ± 0.05	
**Hematological parameters**					
Red blood cells (RBCs 10^6^/mm^3^)	1.57 ± 0.05	1.47 ± 0.26	1.47 ± 0.19		1.43 ± 0.12
White blood cells (WBCs 10^3^/mm^3^)	21.00^b^ ± 0.82	22.00^b^ ± 0.82	25.67^a^ ± 0.94		24.00^a^ ± 1.63
Hemoglobin (Hb g/dl)	10.67 ± 0.47	10.67 ± 0.47	10.33 ± 0.94		10.67 ± 0.47
Packed cell volume (PCV %)	33.67 ± 0.47	34.33 ± 0.47	34.33 ± 2.62		34.00 ± 0.82
Lymphocytes (%)	62.33 ± 1.25	61.00 ± 0.82	61.67 ± 0.94		60.33 ± 2.05
Heterophils (%)	32.70 ± 2.49	34.67 ± 2.36	33.70 ± 1.26		33.89 ± 2.91
H/L ratio	1.92 ± 0.16	1.77 ± 0.12	1.83 ± 0.05		1.79 ± 0.09
Monocytes (%)	3.00 ± 1.41	2.33 ± 1.25	3.00 ± 0.82		3.67 ± 1.70
Basophils (%)	0.67 ± 0.47	0.33 ± 0.47	1.00 ± 0.17		0.67 ± 0.47
Eosinophils (%)	1.30 ± 0.50	1.67 ± 1.25	0.97 ± 0.05		1.33 ± 0.47
**Immunity parameters**					
Phagocytic activity (PA)	19.33 ± 1.25	21.00 ± 0.82	20.67 ± 0.47		20.00 ± 2.16
Phagocytic index (PI %)	1.97 ± 0.12	1.97 ± 0.12	2.00 ± 0.14		1.97 ± 0.17
Antibody titter against NDV; HI	5.67 ± 0.47	6.33 ± 0.94	7.00 ± 0.00		5.67 ± 0.94
Immunoglobulin M; IgM (mg/dl)	23.53 ± 0.17	23.77 ± 0.34	23.30 ± 0.28		23.37 ± 0.21
Immunoglobulin G; IgG (mg/dl)	973.67 ± 2.05	972.67 ± 3.30	969.33 ± 6.85		971.00 ± 2.16
**Lymphoid organs weight (%)**					
Spleen	0.144^a^ ± 0.02	0.081^b^ ± 0.03	0.093^b^ ± 0.03		0.065^b^ ± 0.03
Bursa	0.093 ± 0.05	0.117 ± 0.03	0.133 ± 0.05		0.122 ± 0.05
Thymus	0.187^c^ ± 0.05	0.221^b,c^ ± 0.04	0.315^a^ ± 0.03		0.286^a,b^ ± 0.06

Means not bearing superscripts are not statistically differed (P > 0.05).

^a–c^Means in the same row having different letters are significantly different at (P < 0.05).

### Effect of SiO_2_@AgNPs on immunoglobulin M and G

The data in Table 2 indicates that RBCs, Hb, and PCV% were not significantly affected by the different treatments. However, the WBC counts significantly increased (P < 0.05) in the groups received a dose level of 4 and 8 mg/kg, compared with the control group. Moreover, the percentage of lymphocytes, basophils, eosinophils, monocytes, heterophils, and H/L ratio were not affected (P > 0.05) by the different dose levels of SiO_2_@AgNPs in comparison with the control group. Concerning the immunity parameters (Phagocytic activity (PA), Phagocytic index (PI %), Antibody titter against NDV, immunoglobulin M (IgM) levels, and Immunoglobulin G (IgG)) the analysis of variance showed an insignificant difference (P > 0.05) between the SiO_2_@AgNP levels among the treated and control groups (Table 2). Results in Table 2 shows the difference in the lymphoid organ weight (%) among the groups, i.e., the spleen percentage was significantly affected (P < 0.05) by the different treatments (P < 0.005), and the percentage was decreased when compared with control value. Meanwhile, insignificant (P > 0.05) variation in the relative weights of bursa between all groups. A high significant variation was also observed in the thymus among all groups (P < 0.05), where broilers received SiO_2_@AgNPs at 4 and 8 mg/kg diet showed the highest relative thymus weights compared to other treated and control birds.

### Effect of SiO_2_@AgNPs on blood serum constituents

Based on the analysis of the blood serum constituents of boiler chickens at 5 weeks of age (Table 3), significant variations in albumin (g/dl), aspartate aminotransferase (U/L), total lipids (mg/dl), high-density lipoprotein (mg/l), triglycerides (mg/dl), and creatinine (mg/dl) were observed among all groups, whereas the other blood serum constituents showed insignificant values between treated and control broiler groups. The highest value (3.10 g/dl) of albumin was recorded under a dose level of 4 mg and the lowest value (2.83 g/dl) at a level of 8 mg. Birds treated with 2 and 8 mg of SiO_2_@AgNPs had increased level (P < 0.05) for aspartate aminotransferase (U/L) compared to other treated and control groups (Table 3). The SiO_2_@AgNPs caused a decrease (P < 0.05) in total lipids in broilers supplemented with 4 and 8 mg when compared other treated and control group. For catalase and total antioxidant capacity (Table 3), the analysis of variance detected no significant effect of the SiO_2_@AgNP levels.

**Table 3:** Effect of SiO_2_@AgNPs on blood serum constituents of boiler chickens at 5 weeks of age (n/group = 6).

Items	SiO_2_@AgNPs levels (mg/kg diet) (means ± SD)			
	0	2	4	8
Total protein (g/dl)	5.70 ± 0.16	5.73 ± 0.09	5.77 ± 0.12	5.67 ± 0.12
Albumin (g/dl)	3.03^a^ ± 0.17	2.93^ab^ ± 0.12	3.10^a^ ± 0.08	2.83^b^ ± 0.05
Globulin (g/dl)	2.67 ± 0.12	2.80 ± 0.22	2.67 ± 0.12	2.83 ± 0.12
Albumin/globulin ratio	1.14 ± 0.10	1.06 ± 0.13	1.16 ± 0.07	1.00 ± 0.05
Alkaline phosphatase (μ/l)	1113.30 ± 4.03	1112.70 ± 3.09	1110.70 ± 0.47	1111.00 ± 0.51
Alanine aminotransferase (U/l)	64.00 ± 1.63	64.00 ± 1.63	64.33 ± 1.70	62.33 ± 1.25
Aspartate aminotransferase (U/l)	54.67^b^ ± 0.94	57.67^a^ ± 1.25	52.67^c^ ± 0.94	56.67^a^ ± 1.70
Total lipids (mg/dl)	453.33^a^ ± 16.33	460.00^a^ ± 14.14	430.00^b^ ± 12.47	413.33^b^ ± 22.33
Total cholesterol (mg/l)	212.67 ± 8.26	213.33 ± 3.30	212.00 ± 1.41	210.00 ± 0.82
Low density lipoprotein (mg/l)	42.00 ± 3.56	41.33 ± 0.47	40.67 ± 4.50	40.00 ± 0.82
High density lipoprotein (mg/l)	96.00^bc^ ± 1.00	103.00^a^ ± 2.16	97.67^b^ ± 1.25	95.00^c^ ± 4.71
Triglycerides (mg/dl)	182.67^b^ ± 4.99	190.33^a^ ± 2.05	185.00^b^ ± 0.82	193.00^a^ ± 2.16
Uric acid (mg/dl)	4.33 ± 0.47	4.00 ± 0.82	4.57 ± 0.31	4.50 ± 0.41
Creatinine (mg/dl)	1.23^a^ ± 0.12	1.03^b^ ± 0.05	1.07^b^ ± 0.05	1.10^b^ ± 0.08
Catalase (U/l)	360.00 ± 8.16	370.00 ± 21.60	350.00 ± 8.16	363.33 ± 18.86
Malondialdehyde (MDA) (nmol/ml)	11.67 ± 0.94	10.33 ± 0.47	11.67 ± 0.47	11.33 ± 1.25
Total antioxidant capacity (mg/dl)	1.407 ± 0.06	1.409 ± 0.02	1.405 ± 0.04	1.409 ± 0.03

Means not bearing superscripts are not statistically differed (P > 0.05).

^a^^—^^c^Means in the same row having different letters are significantly different at (P < 0.05).

### Effect of SiO_2_@AgNPs on mRNA expression of IL1β and TNF-α in the muscle tissues

**Figure 2:**
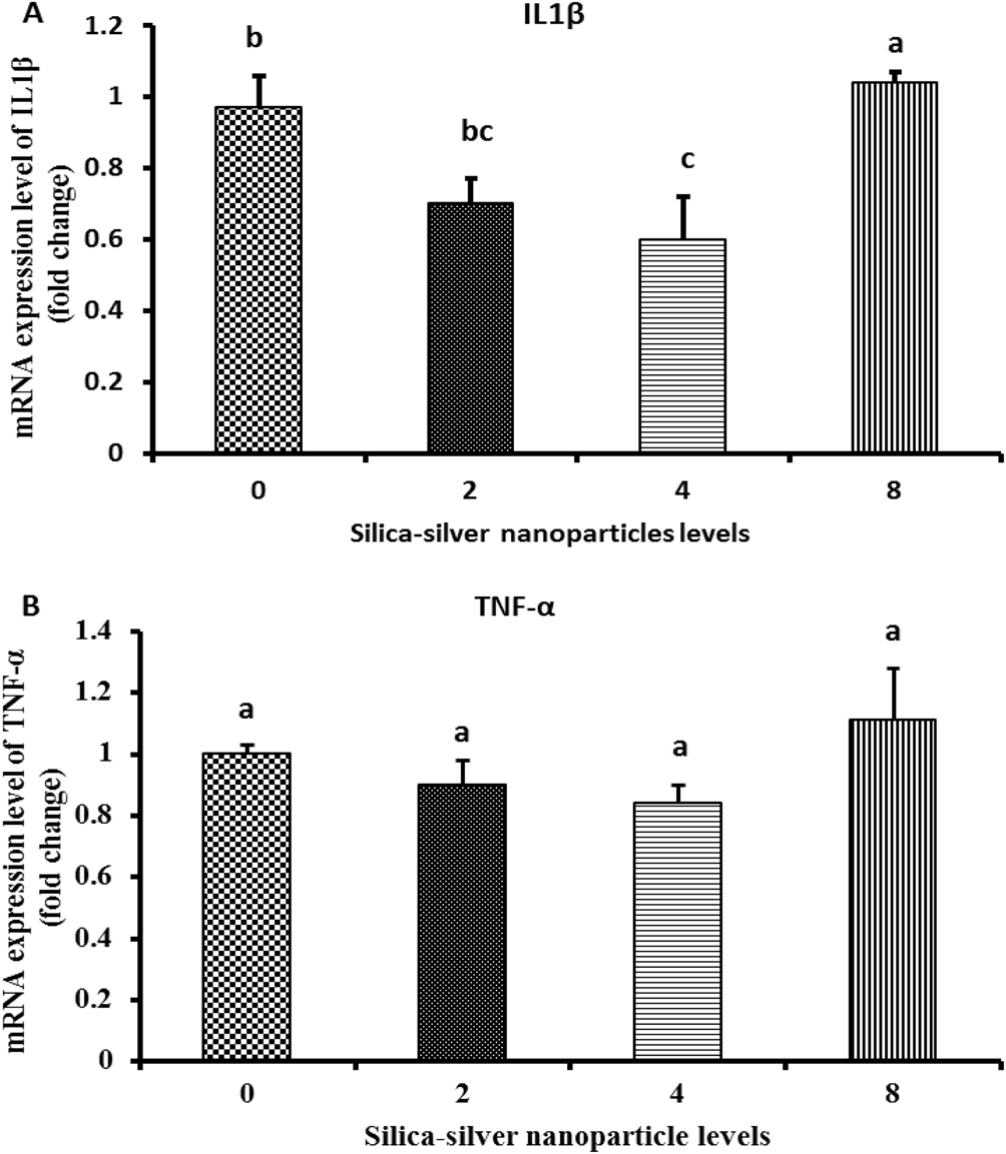
Effect of SiO_2_@AgNPs on mRNA expression of IL1β and TNF-α in muscle tissues. Groups having different letters are significantly different (P < 0.05).

The mRNA expression of interleukin-1 beta *(IL1β*) was examined (Fig. 2). The level of expression in the muscle was higher (P < 0.05) in the group that had received SiO_2_@AgNPs under a dose level of 8 mg/kg compared with the other treatment and control groups, whereas a significant decrease (P < 0.05) was observed in *IL1β* for birds that received silica-silver under a dose level of 4 mg/kg compared with the control group. On the other hand, the mRNA expression of tumor necrosis factor (TNF-α) showed no significant alteration (P > 0.05) between the control and treated groups.

### Histopathological evaluation

#### Liver

**Figure 3:**
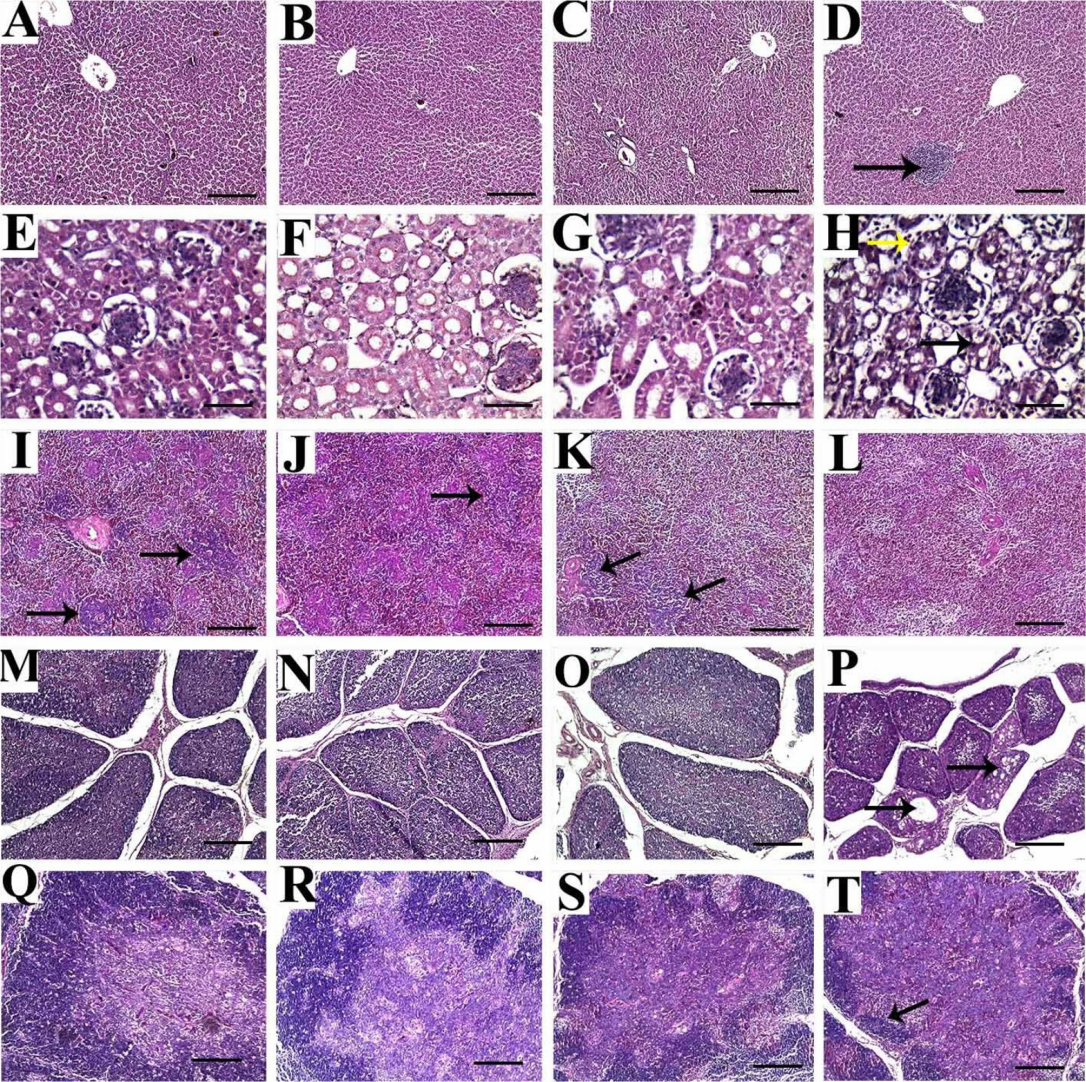
Representative photomicrographs for liver (**A**–**D**), kidney (**E**–**H**), spleen (**I**–**L**), bursa of Fabricius (**M**–**P**) and thymus (**Q**–**T**) of broiler chickens treated with different levels of SiO_2_@AgNPs for 35 days; H&E stain; bar = 100 μm for liver, spleen, bursa, and thymus; bar = 100 μm for kidney. Chickens from control group (**A**, **E**, **I**, **M**, **Q**); chicken treated with 2 mg of SiO_2_@AgNPs (**B**, **F**, **J**, **N**, **R**); chicken treated with 4 mg of SiO_2_@AgNPs (**C**, **G**, **K**, **O**, **S**); chicken treated with 8 mg of SiO_2_@AgNPs (**D**, **H**, **L**, **P**, **T**) showed: healthy normal liver tissue with no specific lesion (**A**–**C**); multifocal accumulations of mononuclear cells (arrow) (**D**); histologically normal structure of renal tubules, renal epithelium, and glomerulus (**E**–**G**); mild intertubular hemorrhage and vacuolization of lining renal epithelium (arrows) (**H**); normal histologic limits of white and red pulps and lymphoid follicles (arrows) (**I**); reduced size of lymphoid follicles (arrow) (**J**); few lymphoid follicles (arrows) (**K**); complete absence of lymphoid follicles (**L**); normal histologic structure of bursa with normal size and number of follicles and normal intensity of lymphocytic populations within medulla and cortex (**M**–**O**); reduced size and number of follicles, reduced medullary cell populations, and frequent cystic structure formation in some bursal follicle (arrows) (**P**); normal histologic structure with normal intensity of medullary and cortical thymocytes and distinct corticomedullary junction (**Q**–**S**); marked loss of cortical basophilic thymocytes and congested medullary vessels (arrows) (**T**).

The control group revealed healthy normal liver tissues with no specific lesions (Fig. 3A). Similarly, the first (2 mg) and second (4 mg) treatment groups showed nearly normal histologic limits of hepatic lobules, central veins, and portal triads (Fig. 3B,C). In contrast, chickens treated with 8 mg of SiO_2_@AgNPs showed an infrequent vacuolization of hydropic type and multifocal accumulations of mononuclear cells (Fig. 3D).

#### Kidney

The kidney tissues from the control and SiO_2_@AgNPs-treated chickens (2 and 4 mg) showed similar histologically normal structures of the renal tubules, renal epithelium, and glomerulus (Fig. 3E–G). However, the third (8 mg) treatment group revealed a mild intertubular hemorrhage and vacuolization of the lining renal epithelium (Fig. 3H).

#### Spleen

The splenic tissues from control group showed normal histologic limits of white and red pulps and lymphoid follicles (Fig. 3I). However, the first (2 mg) treatment group showed a reduced size of lymphoid follicles (Fig. 3J). In addition, the second (4 mg) treatment group showed few lymphoid follicles (Fig. 3K), whereas the third (8 mg) treatment group showed a complete absence of lymphoid follicles (Fig. 3L).

#### Bursa of fabricius

The control group showed a normal histologic structure of the bursa with normal size and number of follicles, prominent corticomedullary junction, and normal intensity of lymphocytic populations within the medulla and cortex (Fig. 3M). Similarly, the first (2 mg) and second (4 mg) treatment groups showed nearly normal histologic limits of the bursa where the histopathologic examination did not reveal signs of necrosis or apoptosis, such as nuclear fragmentation, chromatin condensation, or formation of apoptotic bodies (Fig. 3N,O). On the other hand, the third (8 mg) treatment group showed a reduced size and number of follicles, reduced medullary cell populations, and frequent cystic structure formation in some bursal follicles (Fig. 3P).

#### Thymus

The thymus tissues from control group showed a normal histologic structure with a normal intensity of medullary and cortical thymocytes and distinct corticomedullary junction (Fig. 3Q). In similar manner, the first (2 mg) and second (4 mg) treatment groups showed a nearly normal intensity of cortical thymocytes except in some focal areas that show reduced cortical thymocytes (Fig. 3R,S). However, thymus tissues from birds treated with 8 mg of SiO_2_@AgNPs revealed a marked loss of cortical basophilic thymocytes and congested medullary vessels (Fig. 3T).

### Immunohistochemical evaluation

#### Liver

**Figure 4:**
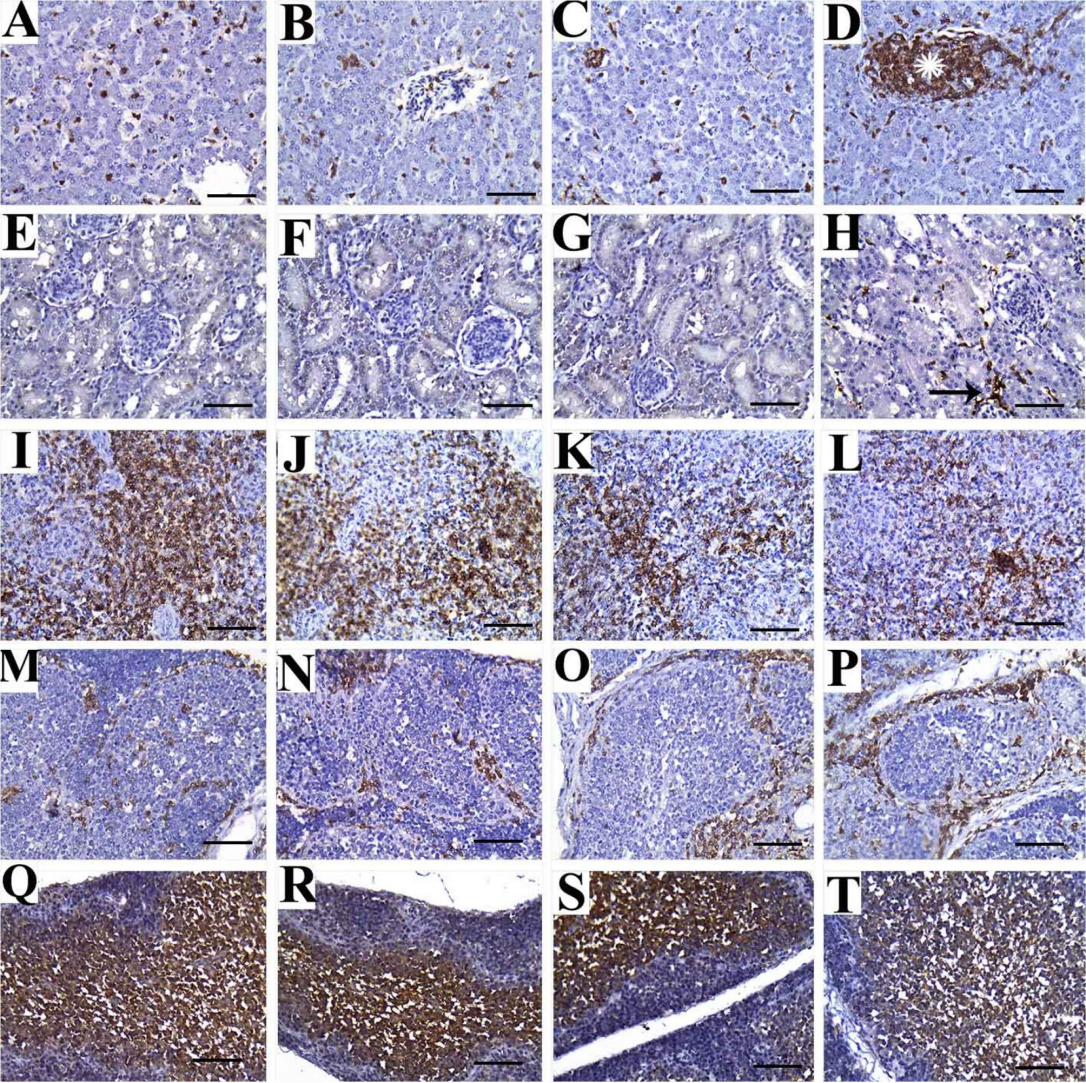
Representative photomicrographs for liver (**A**–**D**), kidney (**E**–**H**), spleen (**I**–**L**), bursa of Fabricius (**M**–**P**) and thymus (**Q**–**T**) of broiler chickens treated with different levels of SiO_2_@AgNPs for 35 days; CD45 immunostaining; bar = 50 μm. Chickens from control group (**A**, **E**, **I**, **M**, **Q**); chicken treated with 2 mg of SiO_2_@AgNPs (**B**, **F**, **J**, **N**, **R**); chicken treated with 4 mg of SiO_2_@AgNPs (**C**, **G**, **K**, **O**, **S**); chicken treated with 8 mg of SiO_2_@AgNPs (**D**, **H**, **L**, **P**, **T**) showed: positive immune expression in form of frequent individual infiltration of leukocytes between hepatocytes (**A**–**C**); multifocal areas of aggregated leukocytes (asterisk) (**D**); negative immune expression against CD45 (**E**–**G**); infrequent interlobular positively stained leukocytes (arrow) (**H**); marked immunoexpression of CD45 at the periarterial lymphoid sheath (PALS) (**I**, **J**); mild to moderate CD45 immunoexpression within PALS (**K**, **L**); mild to moderate immunoexpression of CD45 in the interfollicular areas (**M**, **N**); marked CD45 expression within the inflamed widened interfollicular spaces (**O**, **P**); positive immune staining of CD45 in medullary thymocytes (**Q**–**S**); reduced intensity of immune expression in medullary thymocytes (**T**).

The immune expression of CD45 in the liver tissues of the control group and the first (2 mg) and second (4 mg) treatment groups was observed as a frequent individual infiltration of leukocytes between hepatocytes (Fig. 4A–C). However, the third (8 mg) treatment group showed multifocal areas of aggregated mononuclear leukocytes (Fig. 4D).

#### Kidney

Negative immune expression of CD45 was observed in the renal tissues of the control group and the first (2 mg) and second (4 mg) treatment groups (Fig. 4E–G). However, the third (8 mg) treatment group showed infrequent interlobular positively stained leukocytes (Fig. 4H).

#### Spleen

The immune expression of CD45 in the splenic tissues of the control and treated groups was observed at the periarterial lymphoid sheath (PALS), where T lymphocytes are aggregated in the spleen. With regard to the distribution of CD45 immune expression, a high increase in T-lymphocyte aggregation was observed in the control group and the first treatment (2 mg) group (Fig. 4I,J). On the other hand, the second (4 mg) and third (8 mg) treatment groups showed a marked reduction in immune expression, indicating different degrees of immune depletions in those groups (Fig. 4K,L).

#### Bursa of fabricius

Positive immunoreactivity against CD45 was expressed in the interfollicular areas of the control and treatment groups (Fig. 4M–P). However, it was more frequently expressed in groups that received SiO_2_@AgNPs under doses of 4 and 8 mg, indicating atrophied follicles and inflamed widened interfollicular spaces (Fig. 4O,P, respectively).

#### Thymus

In the thymus, the positive immunostaining against CD45 was evident in the medullary thymocytes, where no significant variations were detected between the control and treatment groups (Fig. 4Q–T). However, a reduced intensity of immune expression was observed in the medullary thymocytes in the third (8 mg) treatment group (Fig. 4T).

### Ultrastructure evaluation of the muscle tissues

**Figure 5:**
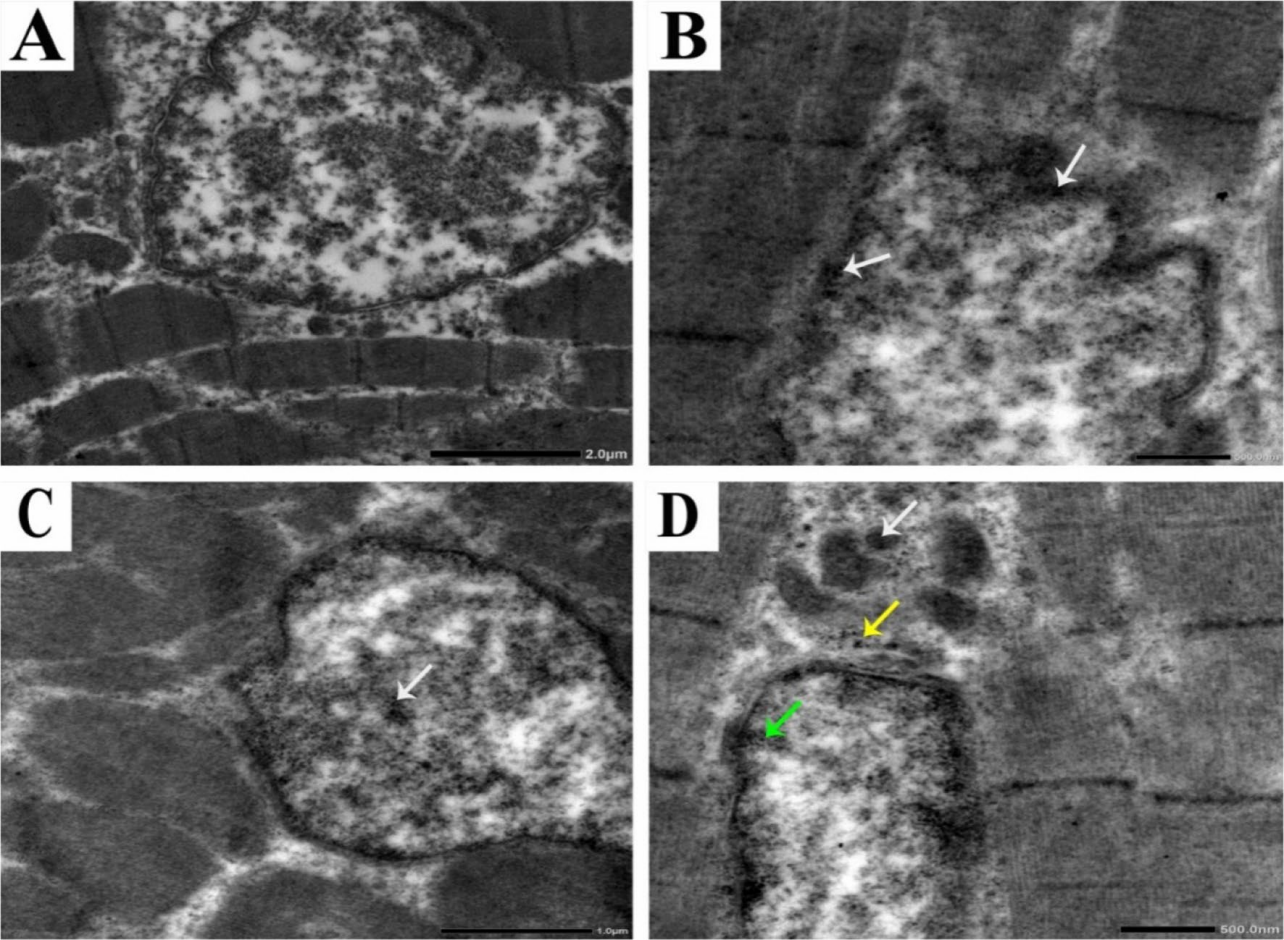
Representative photomicrographs for ultrastructure morphology of muscle tissues from broiler chickens treated with different levels of SiO_2_@AgNPs for 35 days.

TEM was used to confirm the presence or absence of SiO_2_@AgNPs and localize its presence in the breast muscles of chicken compared to the control group. The control group showed a normal structure of the muscles including the nucleus, nuclear envelope, spherical or ovoid-shaped mitochondria with well-developed cristae, filaments, and Z bands (Fig. 5A). However, the muscles of the first (2 mg) and second treatment groups showed an irregular nucleus and abnormal nuclear envelope, disintegrated nuclear chromatin, and aggregations of SiO_2_@AgNP deposits within the nuclear chromatin and near the nuclear envelope (Fig. 5B,C). Moreover, the third treatment (8 mg) group showed an irregular nuclear envelope, disintegrated nuclear chromatin, mild cytoplasmic vacuolization, fragmented mitochondrial cristae, and aggregations of SiO_2_@AgNP deposits within the internal membrane of the mitochondrial cristae, nuclear chromatin particularly the concentrated border lined heterochromatin, and cytoplasm near the nuclear membrane (Fig. 5D).

The control group (A) showed a normal structure of the nucleus, nuclear envelope, mitochondria, and Z bands, whereas the first (2 mg) (B) and second (4 mg) treatment groups showed an irregular nucleus, abnormal nuclear envelope, disintegrated nuclear chromatin, and aggregations of SiO_2_@AgNP deposits within the nuclear chromatin and near the nuclear envelope (arrows). The third (8 mg) (D) treatment group showed an irregular nuclear envelope, disintegrated nuclear chromatin, mild cytoplasmic vacuolization, fragmented mitochondrial cristae, and aggregations of SiO_2_@AgNP deposits within the internal membrane of the mitochondrial cristae (white arrow), concentrated border lined heterochromatin (yellow arrow), and cytoplasm near the nuclear membrane (green arrows).

## Discussion

As shown in Table 2, the effects of the SiO_2_@AgNP levels had no significant variations in terms of the final body weight and body weight gain, feed consumption (g/bird), and feed conversion ratio (g feed/g weight gain) among all groups. These results are consistent with that of Hang and Tra^48^ who reported no differences in the feed consumption (g/bird) among all groups. On contrary, broilers treated with AgNPs exhibited a heavier body weight and high body weight gain than the control group^24,49,50^. Numerous earlier studies showed no enhancement in the growth performance of broilers fed with a diet supplemented with AgNPs^51,52^. The hematological profile in animals is an essential indicator of the physiological or pathophysiological status of the body^53^. Moreover, the results suggest an absence of microcytic hypochromic anemia, which is due to iron deficiency and its improper utilization for the formation of hemoglobin. Hematological constituents reflect the physiological responsiveness of animals to their internal and external environments, which includes feed and feeding^54^. The decrease in the relative organ weights, such as the spleen of boiler chickens supplemented with SiO_2_@AgNPs, might be due to the effect of these nanomaterials on blocking the intestinal absorption of actively transported sugars and amino acids and reduced protein digestibility through the small intestine where mainly enzymatic activity is present as maintained by Vadalasetty et al.^25^. Based on the results of this study, the spleen percentages were decreased in treated groups with different levels with SiO_2_@AgNPs when compared with the control group (P < 0.05) and the bursa (insignificant effect) and thymus showed a significant effect (P < 0.05). These results are partially consistent with that of Abdelsalam et al.^55^. Moreover, the administration of SiO_2_@AgNPs showed a substantial and insignificant effect on all blood parameter values (Table 3). This finding is consistent with that of Ahmadi^56^ who fed broiler chicks with feed supplemented with AgNPs, which exhibited significant changes in the total protein, albumin, and gamma globulin. It was also shown that the AgNPs had insignificant effect on the oxidative stress enzyme activity in the treatment group when compared with the control group. These data indicated that the use of SiO_2_@AgNPs exhibited no harmful effects on the blood serum.

Concerning the effect of SiO_2_@AgNPs on gene expression in chicken muscle tissues, the mRNA expression of IL1β was significantly decreased in birds treated with SiO_2_@AgNPs under a dose level of 4 mg/kg, whereas TNF-α expression was insignificant between the treatment and control groups. Moreover, our results suggest that SiO_2_@AgNPs have an efficient role in reducing the inflammation of broiler muscle tissues particularly under a dose level of 4 mg/kg. These findings are partially consistent with those obtained by Roome et al.^57^ who reported that the administration of SiO_2_@AgNPs in rats significantly (P < 0.05) suppressed the mRNA expression of IL-1β and TNF-α. However, Vadalasetty et al.^25^ found that the expression of *TNF-α* at mRNA level was significantly higher in the liver tissue samples of the broilers supplemented with SiO_2_@AgNPs under a dose level of 50 ppm in drinking water.

In this study, the histopathologic analysis of the liver, kidneys, bursa, thymus, and spleen tissues from the control and treatment groups (at low doses: 2 and 4 mg) showed similar normal histologic limits without specific lesions, which suggest no negative effect of SiO_2_@AgNPs on the histologic structure at such dose levels. However, birds treated with a higher dose level of SiO_2_@AgNPs (8 mg) showed mild to moderate pathologic lesions, such as mononuclear inflammatory infiltrates in the liver and kidney and lymphoid cell depletion in the bursa, thymus, and spleen. Similar findings were reported by Ahmadi et al.^58^ who observed mild necrotic changes and inflammatory cell infiltration in the liver tissues of broiler chickens treated with the highest concentration of SiO_2_@AgNPs. Moreover, Loghman et al.^59^ concluded that treatment with SiO_2_@AgNPs resulted in dose-dependent lesions in the hepatic tissues including hepatic necrosis, focal aggregation of inflammatory cells, and fibroplasias. They also reported hemorrhages in multiple areas and lymphoid depletion in the bursa of Fabricius in SiO_2_@AgNP-treated chickens. These findings may be attributed to the fact that SiO_2_@AgNPs are able to penetrate the cells of various organs including the liver, kidneys, and lymphoid organs by binding to the plasma protein^60^. Inside the cells, SiO_2_@AgNPs are able to diminish the mitochondrial membrane potential with a subsequent increase in ROS regeneration, indicating the role of oxidative stress in SiO_2_@AgNP-mediated cytotoxicity^61,62^. Furthermore, Braydich-Stolle et al.^63^ reported that the SiO_2_@AgNP-induced mitochondrial perturbation increased with the increase in SiO_2_@AgNP concentration. Thus, mitochondria might be considered as sensitive targets of SiO_2_@AgNP cytotoxicity. Hussain et al.^61^ observed that mitochondria had showed an abnormal size and shape in the rat liver cell line (BRL 3A) treated with SiO_2_@AgNPs, which is consistent with our ultrastructural findings. In this study, the ultrastructural examination of the muscle tissues showed that SiO_2_@AgNPs were deposits in the muscle cells either as aggregated dense particles or as singlet. The nanoparticles were located inside the nucleus, near the nuclear membrane, within mitochondria, or deep in the cytoplasm. Interestingly, the fragmentation of chromatin and mitochondrial cristae were noticed particularly in the third (8 mg) treatment group. Similar results were reported by Katsnelson et al.^39^ and El-Habit et al.^41,64^ who concluded that SiO_2_@AgNPs are able to penetrate cells and accumulate within mitochondria, which leads to their destruction. Our results are consistent with previous reports that concluded that the higher the dose level of SiO_2_@AgNPs, the higher the accumulation rate of deposits of SiO_2_@AgNPs^64–68^.

On the other hand, the oxidative stress induced by a high dose level of SiO_2_@AgNPs may subsequently lead to the induction of inflammation and in turn immune suppression; this theory was suggested in our study in relation to the evaluation of the immune expression of CD45^17,18^. CD45 (also known as leukocyte common antigen, as it is used as a common marker for hematopoietic cells except erythrocytes and platelets) plays a key role in the immune system as an essential regulator of T- and B-cell antigen receptor-mediated activation. To the best of our knowledge, no data is available regarding the effects of the dietary supplementation of SiO_2_@AgNPs on the immune expression of CD45 in broilers. In this study, the immune expression of CD45 was upregulated in the liver, kidneys, and bursa of chickens treated with a higher dose level of SiO_2_@AgNPs (8 mg). This upregulation may be attributed to the induced inflammatory response by SiO_2_@AgNPs, which leads to the inflow of mononuclear cells to various tissues, including the liver, kidneys, and bursa. On the other hand, the immune expression of CD45 in cortical thymocytes and the PALS of spleen was decreased in chickens treated with a higher dose (8 mg) of SiO_2_@AgNPs. This finding could be attributed to the adverse effect of the higher dose level of SiO_2_@AgNPs on the immune response of birds as discussed above^17,18^.

## Conclusion

Silver-doped silica nanoparticles (SiO_2_@AgNPs) have been used as an alternative to antibiotics and commonly prepared in powder form. They are obtained in spherical form with a uniform particle size (around 20 nm). The average hydrodynamic diameter of SiO_2_@AgNPs is 51 nm with a polydispersity index (PDI) below 0.24 (below 0.5) confirming the relative narrow size distribution and well dispersibility of the obtained particles. Moreover, SiO_2_@AgNPs have a negative zeta potential (− 33 mv), which is attributed to the presence of a stabilizing agent.

In conclusion, SiO_2_@AgNPs could be considered as a quite safe dietary supplement for broilers due to their anti-inflammatory, antimicrobial, and immune-stimulatory properties. The use of SiO_2_@AgNPs as a safe alternative compound to antibiotic growth promoters is recommended under low dose levels up to 4 mg/kg, since no significant negative effects on growth performance, blood constituents, or histologic structure of internal and immune-related organs are induced in such concentrations. Moreover, the edible muscles showed safe accepted conditions at these low dose levels. In contrast, the dietary supplementation of SiO_2_@AgNPs under a dose level of 8 mg/kg led to mild inflammatory reactions and immune depletion of the bursa, thymus, and spleen. Several serological studies are still needed to investigate the potential effects of SiO_2_@AgNPs at low dose levels on the different types of pathogenic bacteria. Furthermore, the residual effect of SiO_2_@AgNPs on different tissues particularly the edible parts (muscles and livers) needs to be further investigated.
